# Oncological perineal massage in vaginal stenosis and dyspareunia in women with gynecological cancer: a randomized controlled trial

**DOI:** 10.3389/fonc.2025.1680126

**Published:** 2025-11-28

**Authors:** Raquel Pérez-García, Vanesa Abuín-Porras, Isabel Mínguez-Esteban, Daniel Pecos-Martín

**Affiliations:** 1 Department of Nursing and Physiotherapy, Faculty of Medicine and Health Sciences, University of Alcalá, Alcalá de Henares, Spain; 2 Universidad Europea de Madrid, Faculty of Medicine, Health and Sports, Department of Physiotherapy, Villaviciosa de Odón/Madrid, Spain; 3 Grupo de Investigación Fisioterapia y Dolor, Departamento de Enfermería y Fisioterapia, Universidad de Alcalá, Alcalá de Henares/Madrid, Spain

**Keywords:** gynecological cancer, vaginal stenosis, cancer survivorship care, oncological physiotherapy, perineal massage

## Abstract

**Background:**

Dyspareunia and vaginal stenosis are common complications in gynecological cancer survivors. Despite the widespread use of passive vaginal dilator therapy, physiotherapy interventions like oncological perineal massage (MPO^®^) have not been thoroughly evaluated.

**Objective:**

Tis study aims to compare the effectiveness of MPO^®^ perineal massage versus standard passive vaginal dilator therapy in managing dyspareunia, vaginal stenosis, sexual function, and quality of life in women with gynecological cancer.

**Methods:**

A randomized controlled trial involving 35 women (MPO^®^ group: *n* = 18; control group: *n*=17) was conducted. The participants underwent either MPO^®^ massage or passive dilator therapy for 10 weeks, with assessments at baseline (T0), mid-treatment (T1), post-treatment (T2), and 6-month follow-up (T3). Outcomes included pain (VAS), vaginal stenosis (CTCAE 5.0), sexual function (FSM-2), and quality of life (EORTC QLQ-C30).

**Results:**

The MPO^®^ group demonstrated significantly greater reductions in vaginal pain (median VAS: 8.5 at T0 to 0 at T3, *p*<0.001), with improvements also seen in vaginal stenosis (absence/presence at T3: 16/2 vs. 3/14 in controls, *p*<0.001), sexual function (notably lubrication, penetration ease, and satisfaction), and quality of life (QLQ-C30 median score: 32.5 at T0 to 30 at T3 in MPO^®^ vs. 35 to 32 in controls, *p*<0.001).

**Conclusion:**

Oncological perineal massage (MPO^®^) significantly improved pain, vaginal stenosis, sexual function, and quality of life in gynecological cancer survivors compared to passive dilator therapy. These findings support incorporating manual therapy techniques in comprehensive survivorship care in this population.

**Clinical Trial Registration:**

ClinicalTrials [https://clinicaltrials.gov/study/NCT06432998], identifier NCT06432998.

## Introduction

Gynecological cancer refers to malignant tumors of the female reproductive organs, including cervical (CC), ovarian (OC), uterine (UC), vaginal/vulvar (VVC), endometrial (EC), and breast cancer (BC). Globally, these cancers collectively account for approximately 18.9% of all new cancer diagnoses, with breast cancer being the most common (2,296,840 cases), followed by cervical (662,301), uterine (420,368), ovarian (324,603), vulvar (47,336), and vaginal cancer (18,819) (1).

Treatment approaches vary depending on the cancer’s location and characteristics but commonly involve surgery, radiotherapy (RT), brachytherapy (BT), and chemotherapy (CT), either as standalone or combined therapies [OR3]. While these treatments are necessary for disease control, they frequently lead to serious side effects that impact patients’ physical, emotional, and sexual well-being (2–5).

Surgical procedures like hysterectomy, salpingo-oophorectomy, vulvectomy, or pelvic lymphadenectomy affect anatomical integrity and hormone levels while also altering body image, self-esteem, and sexual confidence (6).

Chemotherapy, in particular, disrupts hormonal function by lowering estrogen, progesterone, and testosterone, contributing to ovarian failure and gonadal toxicity (5, 7). As a result, many women experience early menopause as a direct outcome of treatment. The most common reported symptoms are dyspareunia, vaginal dryness, and vulvovaginal burning due to poor lubrication and urinary symptoms (8). Radiotherapy similarly affects ovarian function and estrogen production, and it causes structural changes in the vaginal wall, including vaginal stenosis, atrophy, fibrosis, and loss of elasticity and length (9). It also leads to reduced lubrication due to damage to capillaries and mucosa. This reduced lubrication contributes to inflammation, fibrosis, loss of sensitivity, vulvodynia, and clitoral pain (10).

Sexual dysfunction is among the most prevalent consequences of gynecological cancer treatment and includes a wide array of symptoms: dyspareunia, vaginal dryness, stenosis, vulvar pain, inflammation, anorgasmia, vaginismus, and reduced libido (11). These conditions not only interfere with intimacy but also impair psychological recovery and social well-being (12). Surveys indicate that up to 48% of women report reduced sexual interest, 44% experience less arousal, and 41% report reduced sexual frequency or difficulty relaxing during intercourse post-treatment (6). Dyspareunia, defined as painful sexual intercourse, is particularly common, affecting between 55% and 67% of gynecological cancer survivors. Despite the availability of multiple treatment options such as vaginal dilators, lubricants, moisturizers, local estrogen, and laser therapy, their efficacy is often limited, and adherence rates are poor due to discomfort, embarrassment, or side effects. Consequently, there is a clear need for safe, non-pharmacological, and active interventions (13–17).

Consequently, all of these treatment-related changes collectively contribute to sexual dysfunction and diminished quality of life (6).

Oncological physiotherapy is increasingly important in addressing these complications, emphasizing the need for early and personalized care to preserve function and improve the quality of life; yet, despite its relevance, evidence-based and standardized treatment protocols are lacking (18).

Given the high incidence of gynecological cancer and the common occurrence of dyspareunia, reduced lubrication, and vaginal stenosis, the primary objective of this study was to determine whether the MPO^®^ perineal massage technique offered superior clinical benefit compared to usual care (passive vaginal dilator therapy) in managing dyspareunia and vaginal stenosis among gynecological cancer survivors. We hypothesized that oncological perineal massage (MPO^®^) would result in greater improvements in vaginal pain, reduction of vaginal stenosis, enhancement of sexual function, and improvement in quality of life compared to standard passive vaginal dilator therapy in women treated for gynecological cancer.

## Methods

This randomized, prospective, analytical, and longitudinal clinical trial was conducted in accordance with the CONSORT 2010 reporting guidelines. Prior informed consent was obtained from the participants. The study design was approved by Complejo Hospitalario Universitario de Canarias, Spain (CHUNSC_2023_104), and the trial was prospectively registered (ClinicalTrials: NCT06432998).

### Study population

The participants were recruited through stratified random sampling at the Spanish Association Against Cancer. Eligible participants included women aged 18 to 52 years with a confirmed medical diagnosis of gynecological cancer and associated dyspareunia and vaginal stenosis attributed to oncological treatments such as chemotherapy, radiotherapy, brachytherapy, or hormone-suppressive therapy. All participants provided written informed consent prior to enrollment.

The exclusion criteria comprised refusal to undergo intracavitary treatment, a history of abdominopelvic surgery prior to cancer diagnosis, pre-existing dyspareunia or vaginal stenosis before diagnosis, and menopausal status at the time of diagnosis. Patients in menopausal status at the time of diagnosis were excluded to avoid confounding factors, as menopausal atrophy and dysfunction may pre-exist independently of oncological treatments. Participants were withdrawn from the study if they met the exclusion criteria during follow-up, chose to discontinue participation, or missed more than 25% of the scheduled sessions.

Participants were randomly assigned to either the MPO^®^ group or the control group using a computer-generated randomization sequence created by an independent researcher not involved in participant recruitment or assessment. The sequence was generated using [Microsoft Excel’s RAND function/a random number generator] with a 1:1 allocation ratio and no block or stratification. Allocation concealment was ensured using sequentially numbered, opaque, sealed envelopes (SNOSE), which were prepared by a separate team member. Upon completion of the baseline assessment, the enrolling clinician opened the next envelope in sequence to determine the group assignment.

### Intervention protocol

Participants were randomly assigned to either an experimental group receiving oncological perineal massage (MPO^®^) or a control group receiving passive vaginal dilator therapy. Both groups attended a standardized educational session focused on pelvic floor health and sequelae of cancer treatments.

The experimental group underwent one 50-min weekly session of the MPO^®^ technique during 10 weeks. This intervention included 20 min of internal perineal massage comprising the use, as a massage tool, of vaginal dilators of progressively increasing size (length: 85, 105, 135, and 160 mm; diameter: 22, 27, 32, and 37 mm). The dilators were inserted semicircularly and then mobilized vertically and laterally. Lubricants were not used, although moisturizers were applied when necessary. The participants were instructed to perform the massage independently at home for at least 5 min on non-clinic days.

The control group followed the same schedule and used the same dilators, which were inserted and left in place for 20 min without manual manipulation, as prescribed in the gold standard, usual care, by gynecology specialists for vaginal stenosis. These participants were also instructed to use it daily for a minimum of 5 min.

In the MPO^®^ group, the participants followed a structured progression through four dilator sizes over the 10-week period. While the sequence was standardized, the progression was adjusted based on each participant’s tolerance, with the goal of ensuring comfort and minimizing adverse responses. During the sessions, the physiotherapist inserted the dilators semicircularly and mobilized them vertically and laterally to actively stretch and engage the vaginal tissues. This manual technique was a key component of the intervention. In contrast, the participants in the control group inserted the dilators and held them in place for 20 min without any manual movement, in line with standard passive dilator therapy practices.

Lubricants were not used in either group during the intervention period. This decision was made to avoid masking natural improvements in lubrication and to reduce the risk of irritation or mucosal micro-lacerations, particularly in patients with atrophied tissue. Although lubricants are frequently recommended during intercourse, they do not contribute to mucosal recovery and were therefore excluded.

Vaginal moisturizers, which more closely resemble physiological secretions and provide longer-lasting hydration, were permitted when dilator insertion caused discomfort. Their use was limited to cases of significant intolerance to ensure participant comfort without confounding the assessment of mucosal response and natural lubrication.

Assessments were performed prior to treatment (T0), middle term assessment (T1), post-treatment (T2), and at 6 months post-treatment follow-up (T3) by a blinded assessor, an oncological physiotherapist who specialized in pelvic floor.

### Outcome measures

The primary outcome variables included pain in the vaginal area as perceived by the participant, which was determined with the Visual Analogue Scale (VAS), a 10-cm horizontal line on which the participants marked their perceived pain intensity, with scores ranging from 0 (no pain) to 10 (worst imaginable pain).

Vaginal stenosis was evaluated according to the Common Terminology Criteria for Adverse Events (CTCAE) version 5.0, which classifies the severity of adverse events on a scale from grade 1 to grade 4. For the purpose of the statistical analysis, in this study, the patients were grouped into two categories: absence or presence of adverse events related to vaginal stenosis.

Sexual dysfunction was evaluated using the pain, lubrication, difficulty to penetration, and satisfaction domains of the Female Sexual Function Index 2nd Edition (FSM-2) (19). Each item is scored on a scale from 0 or 1 to 4, with higher scores indicating better sexual function. The FSM-2 evaluates sexual function over the past 4 weeks in women engaging in partnered sexual activity. In this study, this part of the data collection was only applied to sexually active women (MPO *n* = 14; CG *n* = 13).

Quality of life was assessed using the EORTC QLQ-C30 questionnaire, which includes 30 items covering functional domains and symptom scales; global health status and functional scores range from 0 to 100, with higher scores indicating better functioning or quality of life, while symptom scores also range from 0 to 100, with higher scores reflecting greater symptom burden. Raw scores are first calculated as the mean of the items within each domain.

### Sample size calculation

The required sample size was determined using G*Power 3.1 (Heinrich Heine University, Düsseldorf). The calculation was based on the following parameters: a repeated-measures ANOVA model with a between-subjects factor, a two-tailed hypothesis test, an alpha risk of 0.05, a beta risk of 0.20 (corresponding to 80% statistical power), and a 95% confidence interval. The minimally clinically relevant difference (DMCD) for the VAS scale was adjusted to 11 mm based on values reported in the literature in previous studies ranging from 9 to 13 mm (20–22), considering an estimated standard deviation of 15 mm (23). These values yield a standardized effect size of Cohen’s *d*=0.73. For the repeated-measures design, this effect size was converted to Cohen’s *f*=0.49 with *r*=0.5 as the assumed correlation between time points. It was determined that the necessary sample size is 20 subjects per group.

### Statistical analysis

Data analysis was conducted using descriptive and inferential statistics to evaluate the efficacy of oncological perineal massage (MPO^®^) compared to passive vaginal dilator therapy in women with gynecological cancer. All statistical procedures adhered to an alfa level of 0.05 to determine the significance.

Descriptive statistics, including means (standard deviations) or medians (interquartile ranges), were calculated for all continuous variables. Categorical variables were summarized using absolute frequencies (*n*) and percentages (%).

Between-group comparisons at baseline and across time points (post-treatment, final session, and at 6-month follow-up) were performed using independent-samples *t*-tests for normally distributed continuous variables and Mann–Whitney *U*-tests for non-parametric data. Chi-square tests or Fisher’s exact tests were applied for categorical outcomes such as degrees of sexual dysfunction and stenosis severity.

Repeated-measures analyses were conducted to assess within-group changes over time. For parametric variables, repeated-measures ANOVA were applied, with *post hoc* Bonferroni correction where appropriate. For non-parametric variables, Friedman test was used, followed by pairwise Wilcoxon signed-rank tests to examine temporal differences within each group.

All statistical analyses were performed using SPSS (v.27 Armonk, NY, USA: IBM Corp.). Assumptions for parametric tests (normality, homogeneity of variances) were tested and addressed as needed.

## Results

**Figure 1:**
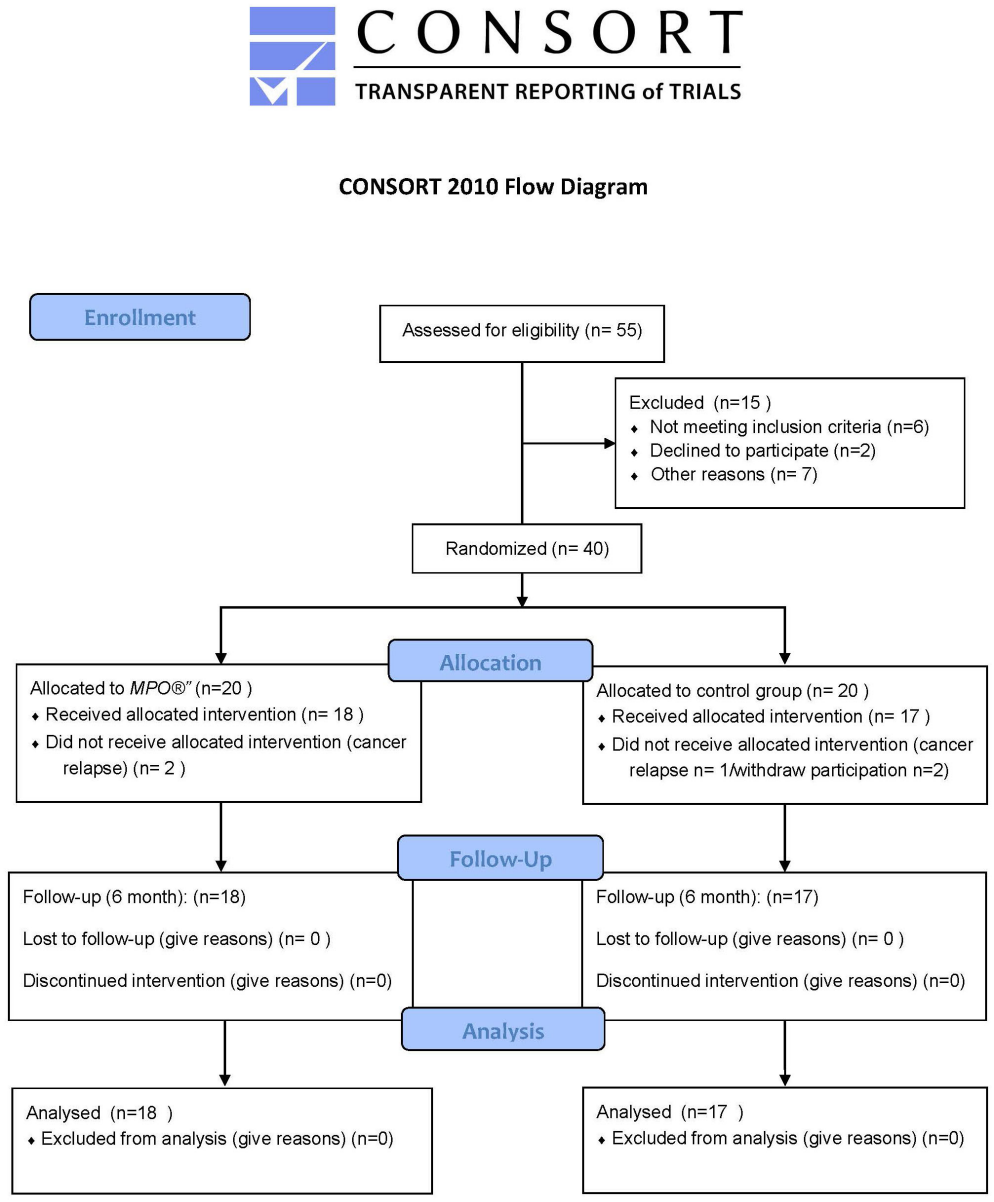
CONSORT flow chart.

A total of 35 participants were included in the analysis, with 18 allocated to the experimental group (oncological perineal massage, MPO^®^) and 17 to the control group (CG; passive vaginal dilator use). A flow chart is shown in **Figure 1**. The demographic data of the sample are shown in **Table 1**.

**Table 1:** Demographic data.

Variable	Total (*n* = 35)	MPO (*n* = 18)	CG (*n* = 17)	*p*-value
Age (years)	41.6 ± 8.2^a^	42.4 ± 8.2 ^a^	40.8 ± 8.3^a^	0.551^b^
Time since intervention (months)	4.0 [3.0–6.0]^c^	3.5 [3.0–5.3]^c^	4.0 [3.0–6.0]^c^	0.490^d^
Oncological treatment				NA
HCT + OOCT + RT	4 (11.4%)	1 (5.6%)	3 (17.6%)	
HCT + OOCT + RT + BT	4 (11.4%)	1 (5.6%)	3 (17.6%)	
HCT + OOCT without RT	3 (8.6%)	1 (5.6%)	2 (11.8%)	
HCT + RT	4 (11.4%)	3 (16.7%)	1 (5.9%)	
HCT + RT + BT	13 (37.1%)	8 (44.4%)	5 (29.4%)	
HCT without RT	4 (11.4%)	2 (11.1%)	2 (11.8%)	
OOCT without RT	2 (5.7%)	2 (11.1%)	0 (0.0%)	
Chemotherapy + RT + BT	1 (2.9%)	0 (0.0%)	1 (5.9%)	

For all analyses, *p*-value <0.05 (for a confidence level of 95%) was considered as statistically significant.

MPO perineal, oncological massage group; CG, control group; IQR, interquartile range; HCT, hysterectomy; OOCT, oophorectomy; RT, radiotherapy; BT, brachytherapy; NA, not applicable due to insufficient sample size for statistical testing.

a Data expressed as mean (standard deviation).

b Student’s *t*-test for independent samples was performed.

c Data expressed as median (interquartile range).

d Mann–Whitney *U*-test was applied.

Baseline VAS pain was slightly higher in the MPO^®^ group (median 8.5) than in the controls (median 8.0; *p*=0.046). At baseline, the MPO^®^ group also reported significantly more pain during intercourse compared to the controls (*p*<0.001). By post-treatment (T2) and at 6-month follow-up (T3), between-group differences favored MPO^®^ (*p*<0.001 at both T2 and T3), with significant group, time, and group × time effects (all *p*<0.001; *η*² = 0.93, 0.92, and 0.82, respectively). Vaginal stenosis improved markedly with MPO^®^ (T3 absence/presence 16/2 vs. 3/14 in controls; *p*<0.001; *φ*=0.72). Sexual function also improved: sexual satisfaction (DDAS_8_T) showed between-group differences at T1 *p*=0.008, T2 *p*=0.001, and T3 *p*=0.004; lubrication (DESERS_3_T) at T1 *p*<0.001, T2 *p*<0.001, and T3 *p*<0.001; and penetration difficulty (DESERS_5_T) at T1 *p*<0.001, T2 *p*=0.002, and T3 *p*=0.006. Quality of life (EORTC QLQ-C30) showed significant between-group differences emerging at T2 *p*=0.041 and T3 *p*<0.001, with significant group, time, and group × time effects (all *p*<0.001).

**Table 2** presents detailed comparisons of the primary and secondary outcomes, including pain, vaginal stenosis, sexual function, and quality of life across the four assessment points.

**Table 2:** Both the intragroup *p*-values (comparison between groups pre-treatment vs. post-treatment for each of the items) and the *p*-values between the four assessments are shown.

Outcomes	MPO (*n* = 18)	CG (*n* = 17)	*p*-value (within groups)	Size effect
VAS (pain in the vaginal area)				
T0	8.5 (1.3)^a^	8 (3)^a^	**0.046** ^b^	
T1	6.2 (1.3)^c^	6.6 (1.8)^c^	0.436^d^	
T2	1 (2)^a^	6 (4)^a^	**<0.001** ^b^	
T3	0 (1)^a^	5 (4.5)^a^	**<0.001** ^b^	
*p*-value (between groups)				
Group effect	<0.001			*η*² (0.93)
Time effect	<0.001			*η*² (0.92)
Group per time effect	<0.001			*η*² (0.82)
Vaginal stenosis (CTACAE 5.0) (absence/presence)				
T0	0/18	0/17	1^e^	*φ* = 0
T1	4/14	0/17	0.1^e^	*φ* = 0.35
T2	15/3	5/12	**<0.001** ^e^	*φ* = 0.54
T3	16/2	3/14	**<0.001** ^e^	*φ* = 0.72
DDAS_8_T (sexual satisfaction) (dysfunction/no dysfunction)				
T0	0/18	0/17	1^e^	*φ* = 0
T1	7/11	0/17	**0.008** ^e^	*φ* = 0.49
T2	14/4	4/13	**0.001** ^e^	*φ* = 0.54
T3	14/4	5/12	**0.004** ^e^	*φ* = 0.48
DESERS_4_T (pain during sexual intercourse) (absence/presence)	MPO sexually active, *n* = 14	CG sexually active, *N* = 13		
T0	6/8	0/13	**<0.001** ^e^	*φ* = 0.51
T1	10/4	0/13	**<0.001** ^e^	*φ* = 0.74
T2	13/1	6/7	**0.013** ^e^	*φ* = 0.51
T3	14/0	7/6	**0.006** ^e^	*φ* = 0.55
DESERS_5_T (difficulty to penetration/stenosis) (absence/presence)				
T0	0/14	0/13	1 ^e^	*φ* = 0
T1	10/14	0/13	**<0.001** ^e^	*φ* = 0.74
T2	14/0	6/7	**0.002** ^e^	*φ* = 0.61
T3	14/0	7/6	**0.006** ^e^	*φ* = 0.55
DESERS_3_T (lubrication) (no dysfunction/dysfunction)				
T0	0/14	0/13	1 ^e^	*φ* = 0
T1	9/5	0/13	**<0.001** ^e^	*φ* = 0.68
T2	14/0	1/12	**<0.001** ^e^	*φ* = 0.93
T3	14/0	4/9	**<0.001** ^e^	*φ* = 0.73
QLQ (quality-of-life score)	MPO (*N* = 18)	CG (*N* = 17)		
T0	32.5 (5.5)^a^	35 (5)^a^	0.157^b^	
T1	32.5 (5.5)^a^	35 (5)^a^	0.157^b^	
T2	31 (3)^a^	33 (5)^a^	**0.041** ^b^	
T3	30 (0)^a^	32 (5)^a^	<0.001^b^	
*p*-value (within groups)				
Group effect	<0.001			*η*² (0.99)
Time effect	<0.001			*η*² (0.62)
Group per time effect	<0.001			*η*² (0.03)

Within-group *p*-values reflect overall changes across time points (T0 to T3) using repeated-measures ANOVA or Friedman test, depending on data distribution. Between-group *p*-values at each time point are reported separately in the “*p*-value (between groups)” column.

T0, pre-intervention; T1, middle term; T2, post-intervention (10 weeks); T3, 6-month follow-up; η², eta partial square; φ, phi.

a Data expressed as median (interquartile range).

b Mann–Whitney *U*-test was applied.

c Data expressed as mean (standard deviation).

d Student’s *t*-test for independent samples was performed.

e Fisher exact test.

**Figure 2:**
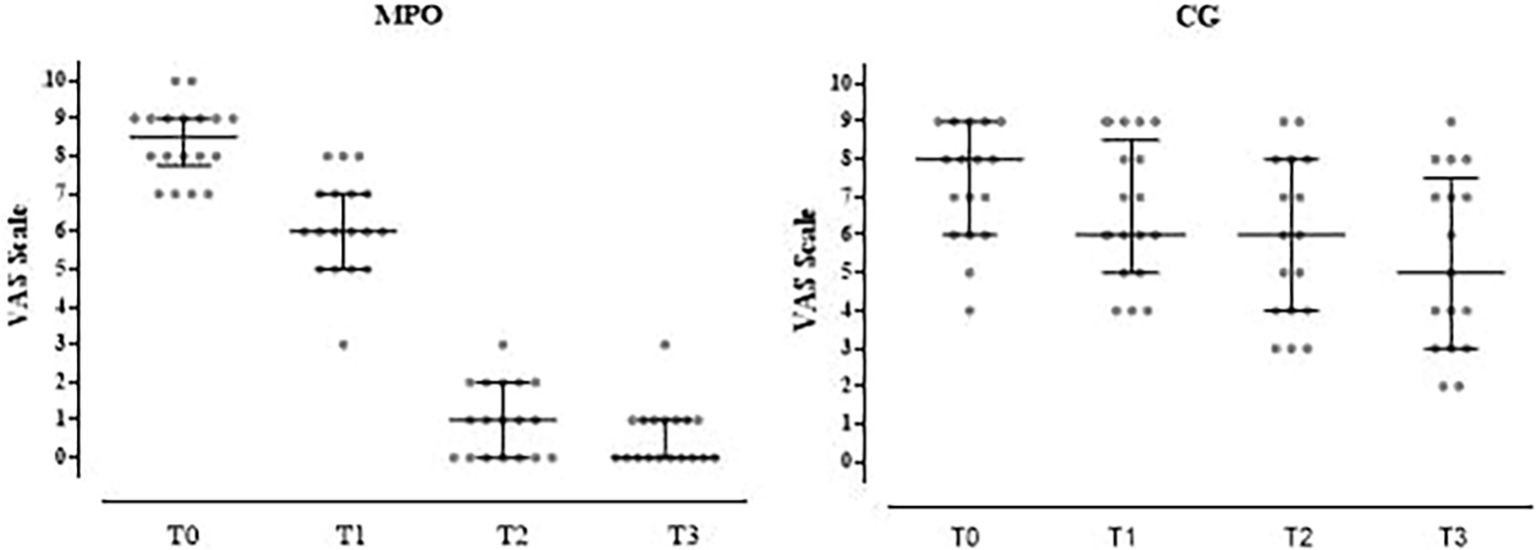
Changes in VAS scores in both groups during the study.

**Figure 3:**
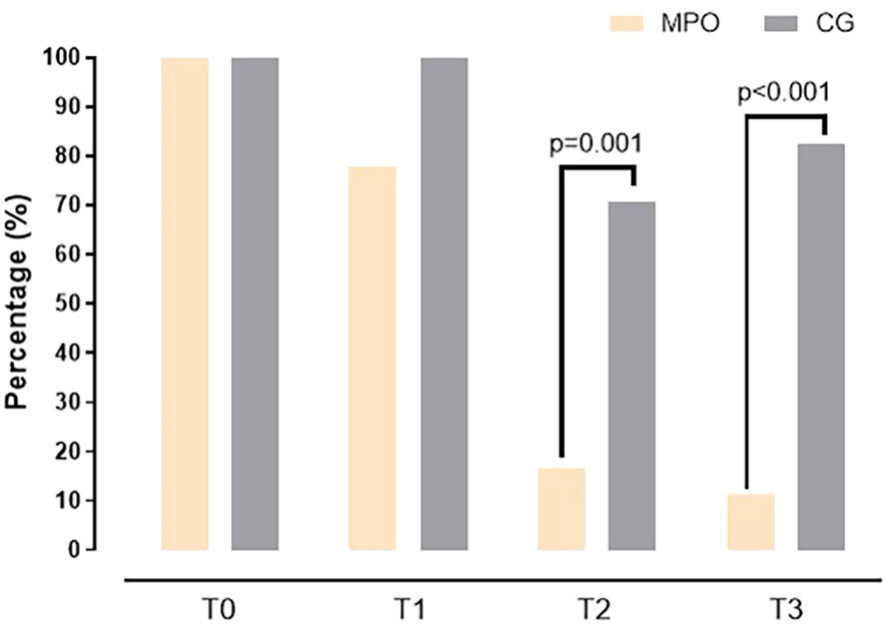
Changes in the presence of vaginal stenosis in both groups during the study.

**Figure 4:**
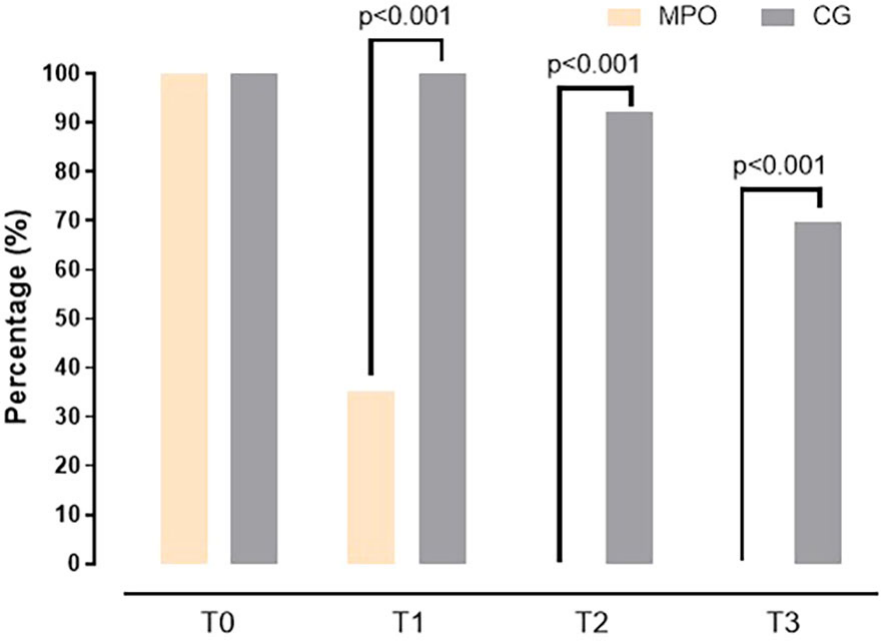
Changes in lubrication dysfunction in both groups during the study.

**Figures 2**–**4** visually depict the evolution of key clinical outcomes throughout the study. **Figure 2** illustrates the marked reduction in vaginal pain scores (VAS) in the MPO^®^ group compared to the control group across all time points. **Figure 3** displays the decline in the presence of vaginal stenosis, with a significant difference favoring the MPO^®^ group by the 6-month follow-up. Finally, **Figure 4** shows the sustained reduction in lubrication in both groups, with differences between groups.

## Discussion

This randomized controlled trial tested the hypothesis that MPO^®^ would produce greater improvements in vaginal pain, reduction of vaginal stenosis, enhancement of sexual function, and improvement in quality of life compared to standard passive vaginal dilator therapy in women treated for gynecological cancer.

The findings of this study support this hypothesis, as the MPO^®^ group demonstrated significantly greater reductions in vaginal pain and stenosis, along with superior improvements in sexual function and quality of life across all time points when compared to usual care.

Participants receiving MPO^®^ experienced a marked decrease in vaginal pain as measured by the VAS despite the fact that the groups were different at baseline, with higher pain scores in the intervention group. Moreover, at baseline, the MPO^®^ group also reported significantly more pain during intercourse than the controls (*p*<0.001). Despite this disadvantage, MPO^®^ participants achieved substantial reductions in pain across follow-up, highlighting the strong effect of the intervention. Significant group differences were observed from post-treatment assessment (T2) onward. By T3, median pain scores in the MPO^®^ group approached 0, indicating substantial clinical benefit (**Table 2**). These improvements are supported by the significant group, time, and group-by-time effects (*η*² > 0.8), indicating a robust treatment effect. Although the MPO^®^ group reported significantly higher pain scores at baseline compared to the controls, our repeated-measures analysis (ANOVA/Friedman) accounted for these differences by focusing on changes over time and group × time interactions. This statistical approach isolates the treatment effect from baseline disparities, ensuring that the observed improvements reflect the true impact of the MPO^®^ intervention rather than regression to the mean or initial imbalance. These findings are consistent with previous studies reporting the effectiveness of physiotherapy-based interventions in gynecologic cancer survivors, including various forms of manual therapy (11, 24, 25). Beyond statistical significance, the magnitude of the observed improvement carries major clinical relevance. The participants in the MPO^®^ group experienced a reduction of vaginal pain scores from severe levels at baseline to almost complete resolution (median VAS=0 at 6 months). Achieving this degree of symptom relief is exceptional in oncological populations, where persistent dyspareunia is often considered a chronic complication. Such outcomes not only improve sexual function but also reduce fear associated with gynecological examinations, strengthen intimate relationships, and enhance overall survivorship quality of life. This highlights the MPO^®^ technique as a potentially transformative intervention in clinical practice.

In terms of vaginal stenosis, the MPO^®^ group also showed superior outcomes. While both groups initially had no adverse events related to stenosis, at 6 months after treatment, 16 of 18 participants in the MPO^®^ group were free from stenosis-related adverse events compared to only three of 17 in the control group (*p*<0.001, *φ*=0.72). This aligns with the hypothesis that active manipulation and stretching of vaginal tissues, rather than passive dilation alone, contributes more effectively to the prevention of fibrosis and atrophy. The superior outcomes of MPO^®^ compared to passive dilators can be understood through several probable physiological mechanisms. First, the active mobilization of tissues enhances local microcirculation, which improves oxygenation and supports mucosal repair without promoting pathological angiogenesis. Second, manual stretching and mobilization may stimulate remodeling of collagen and elastin fibers, thereby reducing fibrosis and restoring elasticity to irradiated or atrophied tissues. Similar processes have been proposed in other physiotherapy interventions targeting pelvic tissues (11, 24). Third, repeated tactile input may contribute to neural desensitization, diminishing hypersensitivity and pain responses while also facilitating psychological adaptation to penetrative stimuli. Finally, the interactive nature of MPO^®^ promotes greater proprioceptive awareness and reconnection with the pelvic region, helping patients regain confidence in their bodies and intimate relationships (26, 27). These mechanisms together provide a plausible explanation for why MPO^®^ was more effective than static dilator use in improving pain, stenosis, and sexual function. This result aligns with those of Nascimento et al. (24), who found that a physical therapy program incorporating perineal massage, progressive dilation, and pelvic floor exercises significantly reversed vaginal stenosis and improved vaginal dimensions in gynecological cancer survivors (24). These findings are consistent with literature indicating the limited effectiveness of passive dilators and the importance of active tissue mobilization in pelvic floor rehabilitation. Araya-Castro et al. (26), in their study, reported problems with adherence to passive dilators related to consequences from cancer treatments, such as forgetfulness, discomfort, feelings of shame, and a lack of coordinated support and information from healthcare providers (26). Similarly, emotional distress, anxiety, negative associations with cancer treatment, and insufficient social or instrumental support were found to significantly undermine the consistent use of dilators among gynecologic cancer survivors (27). In contrast to the adherence challenges reported in these studies, the participants in the MPO^®^ group demonstrated good adherence to the prescribed home exercises. The patients reported that the massage technique was generally well tolerated. Importantly, the participants emphasized that the technique did not provoke pain, which may explain the higher adherence observed compared to conventional passive dilator use. In addition, some participants noted subjective improvements in lubrication and comfort after home practice, which further supported their motivation to continue. These findings suggest that MPO^®^ may be more acceptable to patients and thus more feasible for long-term integration into survivorship care.

Sexual function, assessed through the FSM-2, also improved more notably in the intervention group, particularly in domains related to pain during intercourse, lubrication, penetration difficulty, and satisfaction. These improvements are of particular relevance given the high prevalence of sexual dysfunction in gynecological cancer survivors (28, 29).

Moreover, the vaginal narrowing or vaginismus experienced by these patients have shown to generate significant fear toward routine gynecological examinations, leading women to avoid essential follow-up consultations. This avoidance can result in delayed detection of recurrences or complications, ultimately compromising the patients’ overall health and long-term prognosis (30). Therefore, addressing vaginismus and vaginal stenosis is also a preventive strategy to maintain tolerance for gynecologic exams, as highlighted by Mihulka et al., who found that vaginal stenosis frequently leads to pain and exam intolerance, compromising ongoing surveillance in female cancer survivors (31).

Furthermore, a persistent lack of awareness about pelvic floor disorders contributes to delayed diagnosis and treatment. Many women remain uninformed about the symptoms and clinical implications of these conditions, which leads to postponement in seeking medical attention. Ghandour et al. found that nearly half of women with pelvic floor disorders delayed seeking care for more than 2 years, particularly those with more severe symptoms (32).

Regarding quality of life, MPO^®^ participants reported significant improvements with lower scores, which means a lower symptom burden at the end of the treatment. These gains likely reflect not only physical relief but also psychological benefits from addressing intimacy-related concerns, which are frequently underrecognized in survivorship care. This supports the growing body of evidence advocating for integrative, holistic interventions in oncology rehabilitation (32, 33).

Although statistical comparisons of oncological treatment types were not feasible due to sample size limitations, we conducted a qualitative analysis of treatment distribution to identify potential imbalances between groups. Treatments were categorized based on exposure to radiotherapy (RT) and/or brachytherapy (BT), as these modalities are known to exert greater effects on vaginal tissues.

In the intervention group (MPO^®^), all 18 participants (100%) had received RT or BT as part of their oncological treatment. Similarly, in the control group, 17 out of 17 participants (100%) also had histories of RT and/or BT. This indicates a consistent exposure profile across groups, suggesting that any observed differences in vaginal outcomes are unlikely to be attributed to disparities in treatment intensity. Thus, while statistical testing was not possible, the qualitative equivalence in RT/BT exposure supports the comparability of the two groups regarding this important clinical variable.

### Limitations

Several limitations should be acknowledged. First, two subjects in the MPO and three on the CG were lost due to cancer relapse. Nevertheless, a *post hoc* power analysis showed that this sample yields >80% power. Limitations of recruitment timing made it impossible to previously account for this potential loss. Second, sexual function data were collected only from participants who were sexually active, potentially excluding perspectives from a broader subset of survivors. Moreover, given the nature of the intervention, blinding participants and therapists was unfeasible. Although CTCAE 5.0 is a standardized tool for grading vaginal stenosis, it relies on clinical judgment and may be subject to examiner variability. Finally, the exclusion of postmenopausal women limits the generalizability of the results to a younger subset of survivors. Despite these limitations, this study also has several strengths worth highlighting.

### Strengths

This study presents several notable strengths. First, it is among the few randomized controlled trials evaluating a physiotherapy-based intervention for vaginal stenosis and dyspareunia in gynecological cancer survivors. Second, the use of validated and multidimensional outcome measures (VAS, CTCAE 5.0, FSM-2, and EORTC QLQ-C30) allowed a comprehensive assessment of physical, sexual, and quality-of-life domains. Third, the prospective design with a 6-month follow-up period provided valuable information on the sustainability of treatment effects. Finally, the high adherence and tolerability observed among participants support the feasibility and acceptability of MPO^®^ in this clinical population.

### Future lines

Future studies with larger, multicentric cohorts including menopausal women at the time of diagnosis and longer follow-up periods are warranted to confirm the sustainability of MPO^®^ benefits. Future studies should consider incorporating quantifiable assessments of vaginal dimensions to enhance external validity. Research exploring the integration of oncological physiotherapy into standard gynecological cancer care pathways could further validate its clinical utility.

## Conclusion

Oncological perineal massage appears to be a safe and effective intervention to improve vaginal pain, reduce stenosis, enhance sexual function, and improve the quality of life in women with gynecological cancer. These findings highlight the importance of active pelvic floor rehabilitation and support the inclusion of physiotherapy-based techniques in comprehensive cancer survivorship care. Larger multicenter studies are warranted to confirm these promising results and guide the integration of MPO^®^ into standard clinical practice.

## Data Availability

The original contributions presented in the study are included in the article/**Supplementary Material**. Further inquiries can be directed to the corresponding author.
